# Adverse stimulation of 4-nonylphenol in abnormal reproductive organs of female chickens

**DOI:** 10.18632/oncotarget.21858

**Published:** 2017-10-16

**Authors:** Fenghua Zhang, Peng Yang, Lei Qin, Jie Zhang

**Affiliations:** ^1^ Department of Operating Room, Linyi People's Hospital, Shandong, 276000, China; ^2^ Department of Thoracic Surgery, Linyi People's Hospital, Shandong, 276000, China; ^3^ Laboratory Animal Center, Qiqihar Medical University, Heilongjiang, 161006, China; ^4^ Youth League Committee, Linyi People's Hospital, Shandong, 276000, China

**Keywords:** 4-nonylphenol, reproductive toxicity, environmental endocrine disruptors, chicken, hypothalamo-pituitary-ovarian axis(HPOA)

## Abstract

4-Nonylphenol (4-NP) is a known endocrine disrupting chemical and a persistent environmental contaminant. However, the 4-NP caused mechanism of reproductive toxicity still remains largely unknown in birds. In this study, female chickens (Hy-Line Variety White) were dosed via oral gavage in the early laying period with 0, 50, 100, and 200 mg 4-NP/kg/d for 60 days. Food intake and weight increase were monitored in this organism to investigate chicken growth and development. Moreover, pathological changes of reproductive organs, serum hormone, and mRNA changes on the HPOA were detected. The results showed that gonad development and maturity were retarded in female chickens, and the circulating concentrations of sex hormones were disordered in 4-NP-treated chicken. In 4-NP exposed animals, the mRNA expressions of GnRH and PRLH in hypothalamus and FSH and LH in pituitary were significantly unregulated by 4-NP. In addition, expressions of FSHR and LHR were down-regulated in ovaries of the 4-NP-treatment group, while the levels of stAR, P450scc, P450arom, 3β-HSD, and 17β-HSD were up-regulated in ovaries. Furthermore, expression of ERα in the ovaries of chicken was up-regulated, however, no significant change was observed for ERβ expression. Our results suggest that granulosa cells were an important target and severely disturbed by 4-NP.

## INTRODUCTION

Nonylphenol ethoxylates (NPEOs) are effective non-ionic surfactants, with properties such as emulsification, moisturization, decontamination, and demulsification [1]. It is widely used in numerous industrial fields e.g. for pesticide, leather chemicals, oilfield auxiliary chemicals, and emulsions polymerization [2]. After application as detergents, emulsifiers, dispersants, and humidifiers, about 60% of the NPEOs enter the environment where they are degraded by microorganisms and ultraviolet (UV) light, ultimately becoming nonylphenol (NP), causing pollution in water, soil, and other environmental media [3]. Furthermore, NP has strong lipophilicity, enabling it to accumulate *in vivo* via food chains [4]. Currently, the global annual production of NPEOs amounts to more than 600,000 tons, and China consumes about 100,000 tons per year [3]. In the field of nursing, environmental endocrine disruptors had been confirmed that related to the onset of precocious puberty in children. As the concern about harmful environmental endocrine disruptors (EED) increases, studies on typical EEDs such as NP have received more attention, including investigations on the resulting environmental pollution. Yan [5] lists NP pollution investigation results for China during recent years, revealing extensive 4-NP pollution.

In recent decades, an increasing concern was established for clarifying the toxicological mechanisms of environmental chemicals that cause alterations in the reproductive system of humans and animals [6, 7, 8, 9, 10]. Similar to other effective non-ionic surfactant, NP carries properties that induce endocrine disruption, consequently interfering with various hormonal physiological functions. Numerous reports have suggested that NP might have adverse effects on the reproductive function [11, 12, 13, 14, 15]. In male offspring, a slower maturation of the gonadotrophic system is caused by chronic exposure to NP [16, 17, 18]. In females, it has been shown that NP inhibited the development of the ovary and oviduct in rats, thus causing infertility [19]. It has also been reported that NP induced endocrine disruption and consequently interfered with physiological functions of various hormones [20, 21, 22].

It has been established that 4-NP may impact endocrine activity and notably alter the androgen/estrogen balance [23, 24, 25]. 4-NP is a severe health concern, and numerous studies have been devoted to studying the effects of 4-NP on steroidogenic enzymes that influenced steroid secretion, thus leading to reproductive toxicity [26, 27]. In females, sex steroids are primarily synthesized in the ovaries and are derived from cholesterol through a series of biochemical reactions [28, 29, 30]. However, the effects of 4-NP on the development of ovaries and oviducts, as well as the finely tuned balance between estrogens and androgens is not yet well clarified.

Assessing reproductive toxicity in female animals is challenging, given the complexity of the endocrine system and despite the increasing development of data on its mechanism [30, 31, 32, 33]. To study the bioaccumulation of surfactants and to explore both effects and underlying mechanisms of hormonal balance disruption and developmental abnormalities of ovary and oviduct caused by NP, we utilized 4-NP as test substance, employing female chickens (Hy-Line Variety White) as experimental model. The animals received 4-NP orally and daily in the early laying period with 0, 50, 100, and 200 mg 4-NP/kg/d for a duration of 60 days. The aim of this study was to define the target organ with which 4-NP exerted the effects on the hypothalamo-pituitary-ovarian (HPO) axis and to study the resulting development of ovary and oviduct, thus clarifying the mechanism of 4-NP-induced toxicity in female animals.

## RESULTS

### Effect of 4-NP treatment on food intake and weight increase

First, we measured food intake and weight increase at 6, 12, 18, 24, 30, 36, 42, 48, 54, and 60 days and then added the total food intake and the total weight gains for every experimental group.

**Figure 1:**
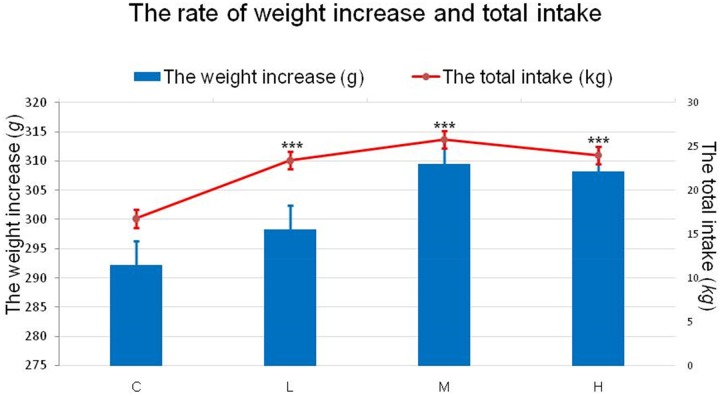
Effects of 4-NP exposure on both rate of weight increase and total intake in chicken The rate of weight increase is represented by the column and the total is represented by the line. The total intake during the experiment significantly increased in the treatment groups, while the weight increase had a slight increase in 4-NP groups. ^***^P < 0.001. C: 0 mg/kg BW; L: 50 mg/kg BW; M: 100 mg/kg BW; H: 200 mg/kg BW.

As shown in Figure 1, a significant increase (*P* < 0.001) was found for food intake between the treatment groups and the control group throughout the entire experiment, demonstrating that 4-NP had an apparent effect on the food intake of chickens. However, the rate of weight gain showed no significant difference (*P* > 0.05).

### Effect of 4-NP treatment on histopathological examination and organ coefficient analysis

**Figure 2:**
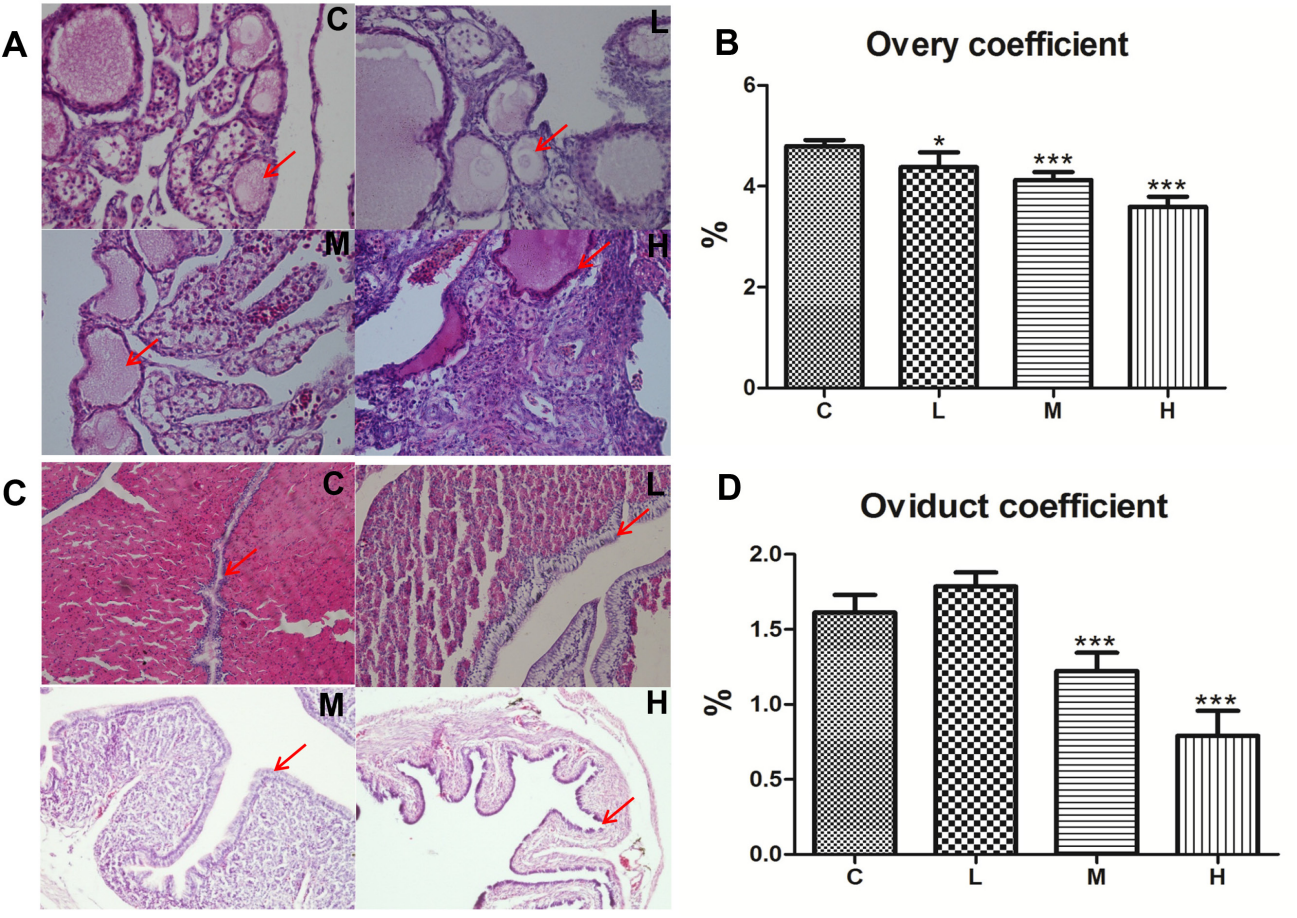
Effects of 4-NP exposure on the development of ovaries and oviducts in chicken **(A)** Progressive changes have been observed in the regression of follicles with increasing dose and suppression in 4-NP-treated chickens (400×). **(B)** The ovary coefficient significantly decreased after 4-NP exposure. **(C)** Photomicrograph showing the transverse section of the shell gland of chicken. Tested birds-mucosal fold displayed low cuboidal luminal epithelium (200×). **(D)** The oviduct coefficient significantly decreased in the M and H groups. ^*^P < 0.05, ^**^P < 0.01, ^***^P < 0.001. C: 0 mg/kg BW; L: 50 mg/kg BW; M: 100 mg/kg BW; H: 200 mg/kg BW.

Next, we investigated the effect of 4-NP on the organ coefficient of ovaries and oviducts in female chicken. Organ coefficient (expressed as tissue-weighting factor) was calculated by dividing the weight of individual chickens by the weight of respective organs of that chicken. The results showed that the coefficient of both ovary and oviduct had decreased except for the L-group of the oviduct, which showed swellings in the anatomy (Figure 2B and 2D).

To further illustrate the damage caused by 4-NP in chicken ovary and oviduct, histopathological changes are shown in Figure 2A and 2C. Photographs of chicken ovaries in different groups are presented in Figure 1A and exhibit a decrease in both number and size of ovarian follicles with increasing dose of 4-NP.

Sexually mature secretory glands were distributed throughout the mucosal folds lined by columnar pseudo-stratified epithelium and had both ciliated and non-ciliated cells [33]. The effect of 4-NP on the oviduct of chickens prior to the hatching period was shown in Figure 1C. The length of the mucosal folds, observed in the transverse section of the shell gland, decreased in the test groups. When compared to the control group, systemic reductions in height and size were noticed in the tubular gland cells and a cellular swelling could be observed in the H-group, which developed into atrophic pathology of epithelial cells.

### Effect of 4-NP treatment on changes in gonadal hormones

**Figure 3:**
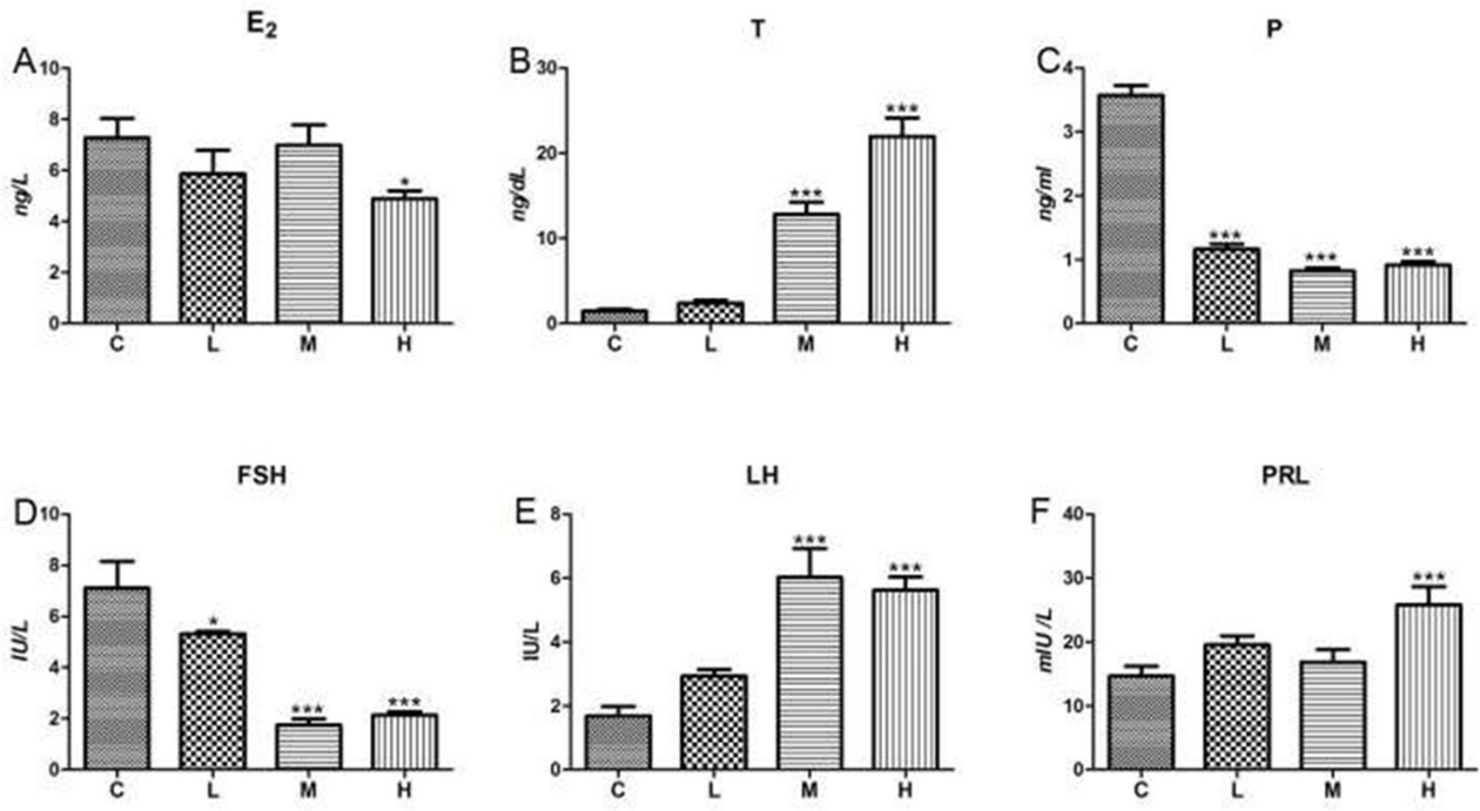
Effects of 4-NP exposure on serum levels of gonadal hormone Gonadal hormone concentration in samples from treatment quails was quantified via RIA kits. **(A)** E_2_; **(B)** T; **(C)** P; **(D)** FSH; **(E)** LH; **(F)** PRL. Data are presented as means ± SD. Compared to controls: ^*^P < 0.05, ^**^P < 0.01, ^***^P < 0.001. C: 0 mg/kg BW; L: 50 mg/kg BW; M: 100 mg/kg BW; H: 200 mg/kg BW.

To explore the dislocation of sex hormone secretion caused by this well-known endocrine disrupting chemical, we detected six gonadal hormones in chicken serum. The oestradiol (E_2_) level of groups with different dosage declined slightly; however, a significant down-regulation was found in the H-group (*P* < 0.05) compared to the control group (Figure 3A). As shown in Figure 3B, 4-NP up-regulated the level of testosterone (T) in the M and H groups (*P* < 0.001). Under the effect of 4-NP, significant differences between control group and treatment groups were found in the level of serum progesterone (P) and serum follicle-stimulating hormone (FSH) (Figures 3C and 3D). The level of luteinizing hormone (LH) showed statistically significant differences in M and H groups (*P* < 0.001) (Figure 3E). The prolactin (PRL) levels among L and M groups were not significantly different as shown in Figure 3F; however, a significant increase was detected for the H-group.

**Figure 4:**
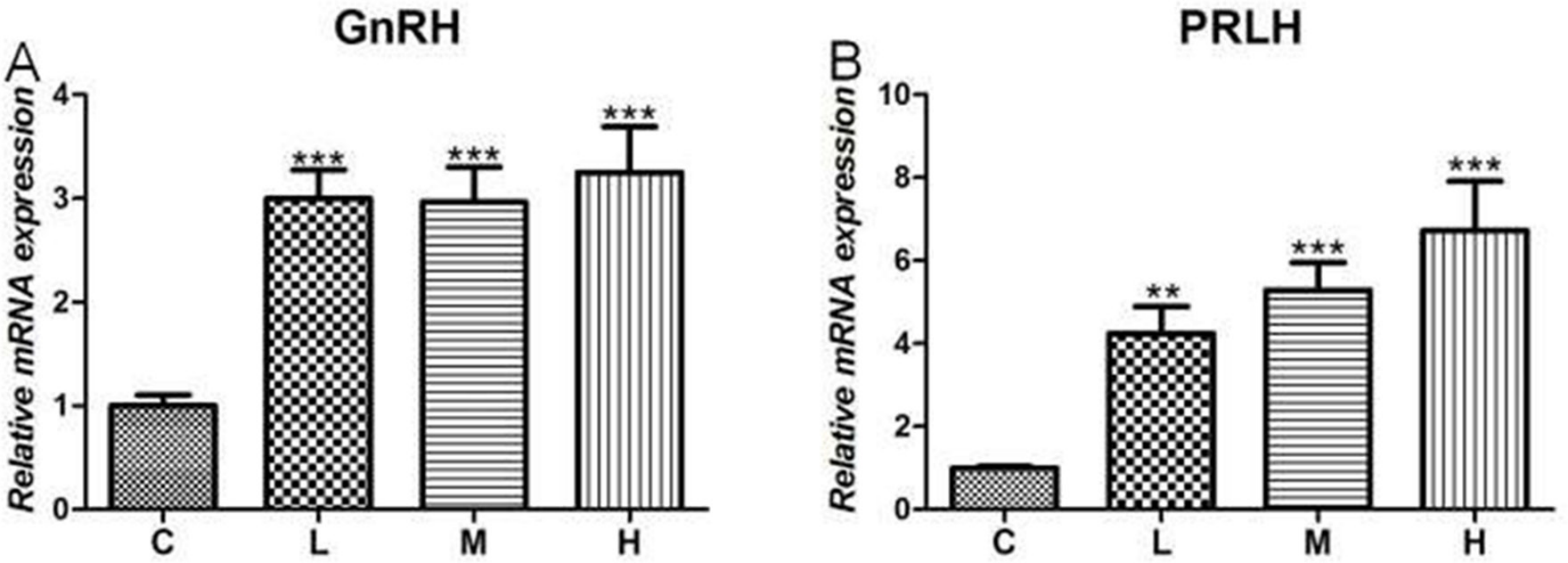
Effects of 4-NP exposure expression of GnRH and PRLH in the hypothalamus **(A)** GnRH in hypothalamus; **(B)** PRLH in hypothalamus. Data are presented as means ± SD. Compared to controls: ^*^P < 0.05, ^**^P < 0.01, ^***^P < 0.001. C: 0 mg/kg BW; L: 50 mg/kg BW; M: 100 mg/kg BW; H: 200 mg/kg BW.

### Effect of 4-NP treatment on the disorder of hypothalamus hormone production

Given that 4-NP directly triggered an imbalance of hormone secretion in the hypothalamus, we next tested changes in relative gene level of 4-NP treatment of the chicken hypothalamus. As shown in Figure 4, the results showed that both gonadotropin-releasing hormone (GnRH) and prolactin-releasing hormone (PRLH) were significantly increased (*P* < 0.001) in the treated groups when compared to C group. Moreover, both GnRH and PRLH mRNA levels were significantly increased in a dose-dependent response in all 4-NP treated groups.

### Effect of 4-NP treatment on gonadotropin in the pituitary gland

**Figure 5:**
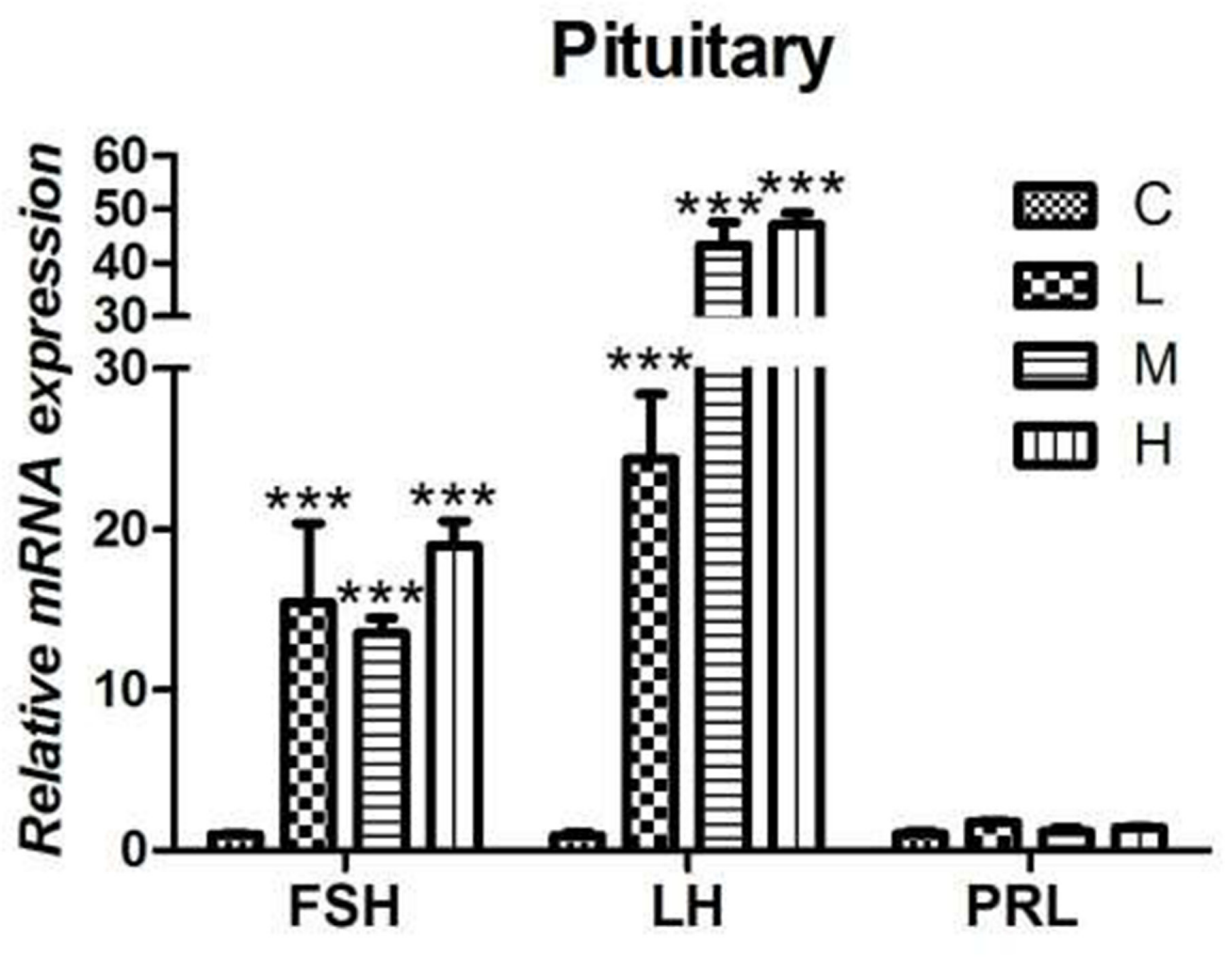
Effects of 4-NP exposure expression of gonadotropins in the pituitary gland The expression of FSH and LH showed a significant increase in all 4-NP treated groups. No significant change was observed in the expression of PRL. ^*^P < 0.05, ^**^P < 0.01, ^***^P < 0.001. C: 0 mg/kg BW; L: 50 mg/kg BW; M: 100 mg/kg BW; H: 200 mg/kg BW.

As shown in the results, the mRNA levels of FSH and LH in pituitary glands of all the 4-NP treated groups were observed up-regulated significantly when compared to C group (*P* < 0.001). However, by the end of the experiment, there was no significant difference of PRL expression level was found between treatment groups and C group as shown in Figure 5.

### Effect of 4-NP treatment on gonadal hormone in ovaries

**Figure 6:**
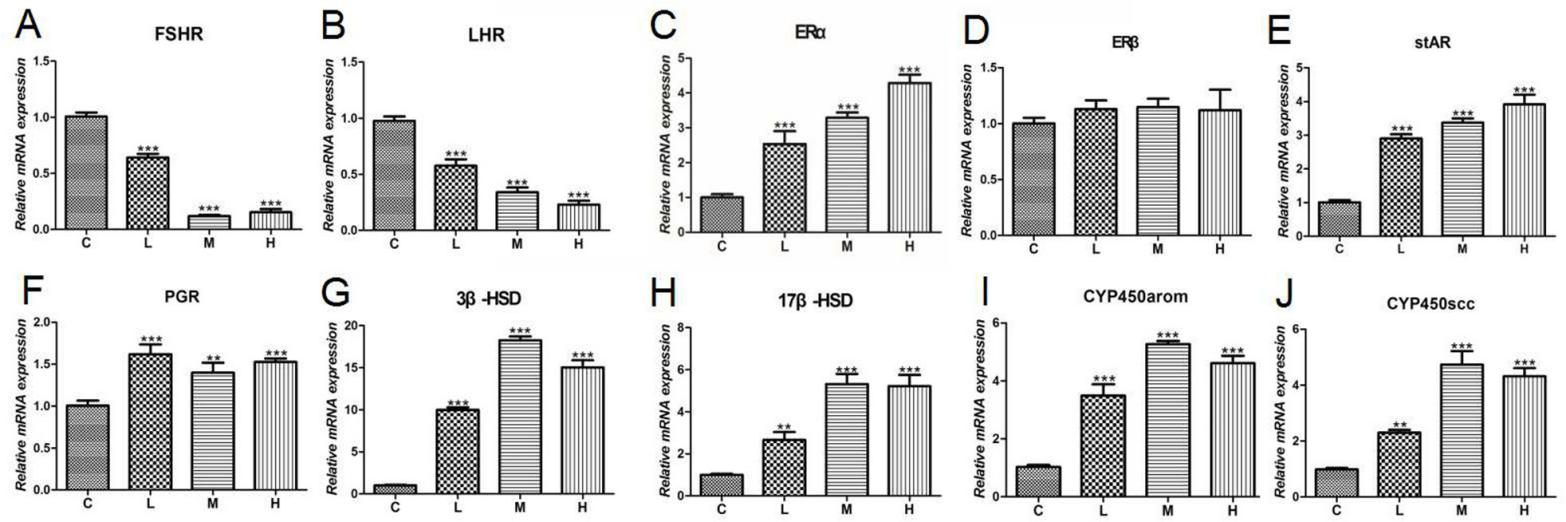
Expression of gonadal hormone-related genes in ovaries of chicken **(A)** FSHR; **(B)** LHR; **(C)** ERα; **(D)** ERβ; **(E)** stAR; **(F)** PGR; **(G)** 3β-HSD; **(H)** 17β-HSD; **(I)** P450arom; **(J)** P450scc. Data are presented as the means ± SD. Compared to controls: ^*^P < 0.05, ^**^P < 0.01, ^***^P < 0.001. C: 0 mg/kg BW; L: 50 mg/kg BW; M: 100 mg/kg BW; H: 200 mg/kg BW.

We next investigated gonadal hormone gene expression levels in chicken ovaries. The effect of 4-NP on follicle-stimulating hormone receptor (FSHR) expression in the ovary is presented in Figure 6A. Compared to C group, a significantly decrease (*P* < 0.001) was detected for FSHR mRNA level in the ovary in chicken exposed to 4-NP. The effect of 4-NP on the luteinizing hormone receptor (LHR) is shown in Figure 6B. The ERα expression levels in 4-NP-treated groups were significantly increased in ovaries (*P* < 0.001) (Figure 6C). Especially, the expression of ERβ mRNA was approximately identical for all groups (Figure 6D).

Steroidogenic acute regulatory protein (StAR) is a rate-limiting protein for the steroid biosynthesis, and the levels of StAR transcripts were significantly increased by 4-NP (Figure 6E) in a dose-dependent response. Simultaneously, the mRNA expressions of progesterone receptor (PGR), 3β-hydroxysteroid dehydrogenase (3β-HSD), and 17β-hydroxysteroid dehydrogenase (17β-HSD) were all significantly up-regulated (*P* < 0.01) (Figure 6F, 6G, and 6H). Figure 6I shows that aromatase cytochrome P450 (P450arom) mRNA expression was up-regulated in response to 4-NP in treatment groups compared to C group (*P <* 0.001). P450 cholesterol side chain cleavage (P450scc) mRNA was also increased in ovaries and a significant difference was observed in every dose group (*P* < 0.001) (Figure 6J).

## DISCUSSION

4-NP has been found to interfere with the reproductive function of many animals [34, 35, 36]. Evidence for the interference of 4-NP due to hormone mimicry has been reported both *in vivo* and *in vitro* [37, 38]. The focus of this study was to clarify toxicological pathways of 4-NP, as an ovarian toxicant, affecting the developmental abnormality of ovary and oviduct in chicken *in vivo*. In the present study, the observed histologic and morphometric changes reflect the arrested development of ovaries and oviducts induced by 4-NP exposure. The developmental abnormalities of ovaries and oviducts are associated with the disruption of gonadal hormone balance and HPO axis in 4-NP-treated chickens. The present study provides new evidence that ovaries and oviducts are the main targets of 4-NP reproductive toxicity.

The widely used 4-NP is a potent endocrine disruptor, altering central nervous system regulation of the reproductive system in females [36, 37, 38, 39]. Numerous studies both in experimental and wild animals suggest that 4-NP can alter normal endocrine and neuroendocrine levels [37, 38, 39]. For instance, exposure to 4-NP has been reported to damage normal gonadal development in Japanese quails [40]. In our result, 4-NP- exposure significantly enhanced the total intake and slightly enhanced the total weight gain, which was also observed in other environmental endocrine disruptors [41]. However, this was the first time that the effect of 4-NP was verified in birds. These effects were also observed for the overt developmental abnormality of ovary and oviduct of treatment groups. The coefficients of reproductive organs were decreased and our result is consistent with that of Zhang [40]. Female reproductive toxicity of 4-NP depended on both dose and duration of exposure in chicken. The results of the present study suggest that the development of ovary and oviduct is more sensitive to 4-NP exposure in sexually maturing female chicken. However, the mechanism of 4-NP-induced developmental toxicity of the female reproductive organs needs further clarification in chicken.

The development of female reproductive organs is regulated via the HPO system through a number of complex feedback loops [42]. These feedback mechanisms are perturbed by ovarian toxicants, especially by ATR [43]. In rodents, repeated exposure to ovarian toxicants produces identifiable histopathological changes in the reproductive tract as well as abnormal hormone secretion [40]. In this study, T, LH, and PRL levels of 4-NP-exposed female chicken were up-regulated to different degrees. Hormone homeostasis had a relative balance to some extent and showed strong self-regulation in birds [44]. Nevertheless, 4-NP plays an important role in disordering the hormone production. E_2_, P, and FSH levels were down regulated.

Our investigation found that the serum FSH level decreased and that the LHR mRNA level in the ovary was also significantly decreased. The result was compared to Harris [20] who found that FSH can stimulate the LHR mRNA level in granulosa cells and can be a novel target of 4-NP in immature granulosa cells. We suspected that due to the damage of 4-NP to the ovarian granulosa cells, both the FSHR and the LHR mRNA level that located in the cell membrane of granulosa cells had a similar decreased. Besides, maybe it's because of negative feedback regulating role of HPO axis that the level FSH and LH in the pituitary was upregulated.

Estrogens act via two types of receptors (ERα and ERβ), which are members of a large super family of proteins, that function as ligand-activated transcription factors [45, 46]. Estrogen signaling is selectively stimulated or inhibited, depending upon a balance between ERα and ERβ activities in target organs [47]. The ERα mRNA level significantly increased; however, no obvious changes were observed in the ERβ mRNA level, suggesting that ERα was the target gene used by 4-NP as the xenoestrogen to disturb the original balance in the ovary.

Moreover, our results illustrate that 4-NP may directly suppress the ovary by stimulating steroidogenic factor expressions, including P450scc, P450arom, 3β-HSD, 17β-HSD, and stAR. Seroidogenesis starts with a transfer of cholesterol into the mitochondria to the site of action of P450scc, which then converts cholesterol to pregnenolone. Pregnenolone is converted to progesterone by 3β-HSD and the conversion of progesterone to androstenedione is then catalyzed by P450arom [12]. We found that all of the levels of expressions of key genes were significantly increased, suggesting that the synthesis pathway of gonadal hormone was stimulated and enhanced by the effect of 4-NP.

However, there are hypotheses about the 4-NP influence on the female chicken hypothalamus. Some studies suggested that 4-NP regulated the target organ and induced negative feedback regulation of hypothalamic hormones [5, 6, 41], which coincides with our results.

Although the pathway of this disruption remains unclear, our results represent new evidence that 4-NP could either directly or indirectly induce developmental abnormalities of ovaries and oviducts.

Due to 4-NP disorder, the experimental inhibition of the formation or action of estrogen in the female chicken and Japanese quail embryos can result in virtually complete phenotypic sex-reversal, such as formation of testis-like ovaries, development of male secondary sex characteristics, lack of oviductal development, and male-like growth of the cloacal gland in response to T [38]. In addition the toxic pathway of 4-NP on the hypothalamic-pituitary-ovarian axis need further exploration.

## MATERIALS AND METHODS

### Ethics statement

In this study, all experiments conducted in animals were in strict accordance with the guidance of the ethical committee for research on laboratory animals.

### Animals and treatments

Chickens (Hy-Line Variety White) aged 100 days and weighing 508.85 ± 13.26 g were purchased from the Baojun breeding center in Harbin, China. 4-NP (C6H5NO3, CAS: 104,405, 98%) was purchased from Sigma. All chickens were housed in cages in an environmentally controlled room (temperature 25-28°C and fluorescent lights provided a photoperiod of 12 h light and 12 h dark). Feed and water were offered *ad libitum* during the entire experiment. After one week of adaptation, the birds were randomly divided into four groups and administered 4-NP once a day orally via gavage for 60 days. The groups were named as follows:

**Table:** 

Groups	4-NP	Solvents
C	0mg/kg	corn oil
L	50mg/kg	corn oil
M	100mg/kg	corn oil
H	200mg/kg	corn oil

The birds were monitored daily for clinical signs. Both the total body weight gain and total intake were measured. At the end of the experiment, birds were fasted before the day of sacrifice, their fallopian tubes and ovaries were quickly removed and weighed, after which they were immediately kept at −80°C until further use. Their hypothalamus and pituitary gland were carefully dissected and then immediately frozen at −80°C until further use. The blood was collected from the heart of each bird and centrifuged at 3,000 rpm for 10 min, thus obtaining the serum for hormone analysis.

### Histopathological studies

Oviducts and ovaries were washed in cold saline and soak dried on filter paper. A portion of the organ was fixed in 10% buffered formalin and embedded in paraffin. Sections of 5 μm thickness were cut and stained with both hematoxylin and eosin for microscopic examination.

### Hormone analysis

To identify 4-NP-induced changes in circulating concentrations of reproductive hormones, serum concentrations of E2, P, FSH, LH, PRL, and T were determined. All six reproductive hormones were determined via ^125^I Radioimmunoassay (RIA) Kits (HAT CO., LTD., China) according to the manufacturer's protocol. Radioactivity was determined with an automatic gamma counter. All samples were run in duplicate in a single assay to avoid inter-assay variation.

### RNA purification and quantitative real-time PCR

Total mRNA was extracted from hypothalamus, pituitary gland, and ovary using the RNAout reagent (Beijing Tiandz, Inc., China) according to the manufacturer's instructions. The first cDNA strand was synthesized using Oligo (dT) primers and transcript reverse transcriptase (Beijing TransGen Biotech Co. Ltd., China). The primers for the real-time amplification of relative cDNAs were designed using Oligo 7.22 software (Molecular Biology Insights, Cascade, CO) based on the deposited sequences in GenBank and primers are presented in Supplementary Table 1. Quantitative real-time PCR (qRT-PCR) was conducted with a fast real-time PCR system (ABI PRISM 7500 real-time PCR system (Applied Biosystems, CA). Triplicate samples were assessed for each gene of interest, and β-actin was used as control gene. Relative expression levels were determined via the 2^−ΔΔCt^ method. Relative expression levels of genes were calculated respectively. C group was used as endogenous control.

### Statistical analysis

The data were statistically analyzed using GraphPad Prism 5.1 (GraphPad Software Inc., USA). One-way analysis of variance (ANOVA) and least significant difference (LSD) post hoc tests were utilized to analyze the data. Differences between the means of data were analyzed with Dunnett's multiple comparisons test and the paired T-test, which was utilized to determine the effects of ATR. The results were expressed as mean ± S.D. of different groups. Significant differences of all data were shown via ANOVA for each experiment. P < 0.05 was considered significant.

## SUPPLEMENTARY MATERIALS TABLE


