# Supplementary material for: Adverse stimulation of 4-nonylphenol in abnormal reproductive organs of female chickens
Source: Oncotarget. 2017 Oct 16;8(66):110029–38. doi: 10.18632/oncotarget.21858 (PMC5746362; doi:10.18632/oncotarget.21858)
Supplement: Supplementary file 1 [file oncotarget-08-110029-s001.pdf]

Adverse stimulation of 4-nonylphenol in abnormal reproductive organs of female chickens

SUPPLEMENTARY MATERIALS

Supplementary Table 1: Oligonucleotide primers used in RT-qPCR analysis for gonadal hormone genes expression in HPO axis of gallus

Gene	ReSeq ID	Sequence of primer	PCR products
β-actin	L08165	Forward:5'- CCGCTCTATGAAGGCTACGC -3'<br>Reverse:5'- CTCTCGGCTGTGGTGGTGAA -3'	128bp
GnRH	AY635931	Forward:5'- GTTCTCCTGTTTACCACGTCT -3'<br>Reverse:5'- TTCCACCAGATTTTCAGCGTTC -3'	99bp
PRLH	NM_001082419	Forward:5'- AGGAACCCAGACATCGACCC -3'<br>Reverse:5'- CCGTCGCCCGAATCTCCC -3'	72bp
FSH	AB086952.1	Forward:5'-TGGTGCTCAGGATACTGCTTCA-3'<br>Reverse:5'-AGATTCAGGATGGTCACCGCAG-3'	132bp
LH	S70834	Forward: 5'-AACCCGTTTACCGCAGCC-3'<br>Reverse: 5'- GCAGCCCCATAGATCCCAAC-3'	92bp
PRL	AB162003	Forward: 5'-GGTTCATTCTGGCGATGGTGGA-3'<br>Reverse: 5'-CGGAAGCAATGCAGCAGGTTGT-3'	120bp
$P450_{SCC}$	NM_001001756	Forward: 5'-AGCACTTCAAGGGACTGAGC-3'<br>Reverse: 5'- ACTTGGTCCCAACTTCCACC-3'	147bp
P450arom	S46949	Forward: 5'- TGATGACATGCCAAACCTCA-3'<br>Reverse: 5'- CCAGCTTGTGCATACGTCCA-3'	173bp
3β-HSD	FJ607242	Forward: 5'- CACAGAAAGCCAGACGCATC-3'<br>Reverse: 5'- AAAGCCCAGCTCCTTGGTCA-3'	107bp
17β-HSD	AB002410.1	Forward: 5'- CAGCTCTACCGCCGCTACCTG-3'<br>Reverse: 5'- GCGATGGCCTCCAAGAACACC-3'	98bp
ERα	AF442965.1	Forward: 5'-TGTCCTGCCACTAACCA-3'<br>Reverse: 5'- TTTCATCATTCGCCCAC-3'	138bp
ERβ	AF045149	Forward: 5'-AACCCGTTTACCGCAGCC-3'<br>Reverse: 5'- GCAGCCCCATAGATCCCAAC-3'	96bp
stAR	NM_204686	Forward: 5'- AAGCCCTGCAGAAATCACTC -3'<br>Reverse: 5'- TCAGCACTTTGTCTCCGTTG -3'	87bp
PGR	AB265142	Forward: 5'- GTCAGACTCCAAGTTAGCCA -3'<br>Reverse: 5'- GGAATTAGCCACAACACCCT -3'	177bp
